# The burden of mental disorders attributable by cocaine use: Global Burden of Diseases Study in Brazil, 1990 and 2019

**DOI:** 10.1590/0037-8682-0320-2021

**Published:** 2022-01-28

**Authors:** Rayce dos Santos Crepalde, Cecília Silva Costa Bonadiman, Deborah Carvalho Malta, Mohsen Naghavi, Ana Paula Souto Melo

**Affiliations:** 1 Universidade Federal de Minas Gerais, Faculdade de Medicina, Programa de Pós-Graduação em Saúde Pública, Belo Horizonte, MG, Brasil.; 2 Universidade Federal de Minas Gerais, Escola de Enfermagem, Belo Horizonte, MG, Brasil.; 3 Institute for Health Metrics and Evaluation, Seattle, WA, USA.; 4 Universidade Federal de São João Del Rei, Faculdade de Medicina, Divinópolis, MG, Brasil.

**Keywords:** Cocaine, Descriptive epidemiology, Mortality, Disability adjusted life years, Brazil

## Abstract

**INTRODUCTION:**

Brazil is an important consumer market for cocaine. However, the consequences of this consumption and the pattern of distribution of the estimates are still poorly studied in the Brazilian states. The Global Burden of Disease study - 2019 (GBD-2019) has enabled us to describe and analyze indicators of mental disorders (MD) attributable by cocaine use in Brazil and its states, in 1990 and 2019.

**METHODS:**

A descriptive study of the burden of cocaine use disorders, using prevalence, age-standardized mortality rate (ASMR), years of life lost (YLL) due to premature death, years lived with disabilities (YLD), and disability adjusted life years (DALY), which accounts for YLL+YLD.

**RESULTS:**

Brazil ranks 8^th^ as DALYs due to cocaine use disorder in the world (42.83/100.000; 95% uncertainty intervals [95% UI]: 35.28 to 61.43). Significant increases have occurred in the age-standardized rate prevalence (ASRP), ASMR, DALY, YLD, and YLL, in Brazil and its states, between 1990 and 2019. The ASRP in 2019 was 2.7-fold higher for men (278.60/100.000; 95% UI: 208.20 to 374.39) in comparison to women (104.01/100.000; 95% UI: 76.70 to 143.02). There is a predominance of YLD in the composition of DALYs; however, the YLL had the biggest increases between 1990 and 2019.

**CONCLUSIONS:**

The high rate of DALYs and the increase in mortality rates show the need to scale up effective interventions to prevent and reduce the burden of disease attributable to cocaine use disorder, which is a preventable cause of death and disability.

## INTRODUCTION

In 2017, according to one estimate, 0.4% of global population aged 15-64 years used cocaine and the prevalence has increased in North America and in some South American countries^1^.

Brazil is the world's second largest consumer market for cocaine^2^. Addressing the use of cocaine, especially crack cocaine, is considered as a matter of “health emergency”^3^. The Third National Survey on Drug Abuse by the Brazilian Population revealed, in 2015, the most used illicit drug was cannabis: 7.7% of population had used it at least once, 2.5% in the previous 12 months, and 1.5% in the last 30 days. The second most frequently used drug was cocaine, with a life time prevalence of 3.1%, 0.9% in the previous 12 months, and 0.3% in the previous 30 days^4^. Moreover, it was estimated, in 2012, 0.81% of population from the state capital cities and the Federal District had used it regularly (25 days out of the six months before the study), either as crack cocaine or similar products (cocaine paste, *merla*, oxi)^5^. The use, abuse, and dependence of cocaine and crack are related to increased prevalence of infectious diseases, clinical and psychiatric morbidity, premature deaths, violent behavior, unprotected sex, family conflicts, among other problems^2^ ^,^ ^6^ ^-^ ^8^.

The Global Burden of Disease (GBD) Study is an important tool to provide subsidies for political decision-making by means of estimates, which are comparable between different regions, countries, and populations. The GBD study measures the burden of diseases by years of life lost (YLL) due to premature death and years lived with disability (YLD); the sum of both is the Disability Adjusted Life Years (DALY); in other words, DALY=YLL + YLD^9^.

Considering the magnitude of data on use of cocaine in Brazil, the objective of this study is to describe the estimates of dependence prevalence, mortality, YLL, YLD, and DALY related to the disorders attributable by use of cocaine in Brazil and its states, according to sex and age groups, in 1990 and 2019.

## METHODS

A descriptive study of mental disorders (MD) related to cocaine use was conducted with individuals aged 15 years and older, using the estimates of the GBD 2019 as the main source of data. All GBD 2019 analyses followed the Guidelines for Accurate and Transparent Health Estimates (GATHER)^10^. The GBD 2019 follows the principles of the PRISMA guidelines, as mentioned by Moher *et al.* ^11^. Data extraction complies with the STROBE guidelines^12^. The estimates used by the GBD 2019 are available at the website of the Institute of Health Metrics and Evaluation (IHME), at http://ghdx.healthdata.org.

In the GBD 2019, MD attributable by use of cocaine were defined according to the International Classification of Diseases, 10^th^ Revision^13^ (ICD-10), and the Diagnostic and Statistical Manual of Mental Disorders, version 4 (DSM-IV)^14^. The ICD-10 codes used by the GBD 2019 for the study estimates were F14-F14.99 and T40.5^15^. In the GBD 2019, as well as in the ICD-10, the type of cocaine use are not differentiated; therefore, the term “cocaine” will be generalized to refer to all forms the drug is consumed, either in snorted, injected, or smoked.

For Brazil, the GBD 2019 estimates of prevalence, incidence, and mortality excess, associated with each disorder attributable by illicit drugs, were based on data from the Mortality Information System (SIM, in Portuguese), the Notifiable Disease Information System (SINAN, in Portuguese), as well as from epidemiological surveys and scientific articles. The citations of all sources are found at http://ghdx.healthdata.org/geography/brazil.

The GBD 2019 study uses the ICD principle of attributing an underlying cause of death to each death; in other words, the cause that triggered the series of events resulting in death^16^. Thus, deaths having cocaine as their underlying cause of death will be used to estimate the mortality rates. However, to qualify the mortality rate estimates, the GBD 2019 adjusts for garbage codes, or non-specific causes (causes of death attributed to motives that cannot be considered as such). In this sense, in the GBD 2019, the ICD-10 codes of accidental poisoning (X40-44) were redistributed to mental and behavioral disorders due to psychoactive substance use, using an algorithm developed through the analysis of multiple causes of death in the United States of America (USA), Mexico, Brazil, Taiwan, Italy, Colombia, Australia, and several European countries, from 1980 to 2017^15^. The main proposition of this algorithm is the fatality dominance of the illicit substance when multiple causes of death are considered. According to redistribution, when opioids are present as one of the intermediate causes of death by accidental poisoning (X40-44), they dominate the other categories of drugs, followed by cocaine, amphetamines, and psychoactive and psychedelic drugs (which are equally fatal), followed by alcohol and, finally, cannabis^15^. However, for the 2019 estimates, these algorithms were revised, and seven models were proposed, according to distinctions in drug consumption in each country. The models proposed for the USA show a dominance of opioids, followed by cocaine and other drugs. In Model 7, specific for Brazil, cocaine shows a dominance in redistribution (nearly 80%), followed by other drugs, opioids, and alcohol^17^.

The causes of death were modelled according to the Cause of Death Ensemble Model (CODEm), an analytical tool that tests different statistical models of causes of death, generating a combined set of models that offer a better predictive performance^18^ ^,^ ^19^. To assure the number of deaths does not exceed the total number of deaths estimated by the GBD 2019, a correction technique, known as CoDCorrect, was applied. This technique guarantees the estimate of number of deaths for each cause does not add up to more than 100% of deaths, in a given year^9^.

In the GBD 2019, the burden of disease is measured by the DALY, which is the sum of YLL by premature death and the YLD^9^. The YLL represent the effect of premature deaths within the population and were calculated by using the standard methods from the GBD 2019, in which each death is multiplied by the standard life expectancy for each age group. To produce age-standardized rates of mortality and of YLL (per 100,000 inhabitants), a direct standardization method was used, considering the global population developed for the GBD 2019 as a standard. Premature deaths stand out by applying a greater weight to deaths that occurred in younger age groups^20^.

To calculate the YLD, the prevalence of the MD attributable by cocaine use is multiplied by a disability weight associated with it. The disability weight reflects the severity of health loss associated with the MD attributable by use of illicit drugs, in a scale of 0 to 1^15^. The disability weights were estimated for each level of severity of MD attributable by cocaine use, and are classified as asymptomatic (weight equal to zero), light (weight equal to 0.116, 95% CI: 0.074 - 0.165), and moderate/severe (weight equal to 0.479, 95% CI: 0.324 - 0.634)^15^.

Epidemiological data obtained from the literature was modeled using the DisMod-MR 2.1 software, a Bayesian meta-regression tool that pools datapoints from different sources and adjusts for known sources of variability (differences in definitions of case, methodology, and sample), to produce internally consistent estimates of incidence, prevalence, remission, and mortality excess^15^.

The GBD models the prevalence results of cocaine use and dependence using the DisMod-MR to obtain the dependence prevalences^12^. Therefore, in the results of this study, the term “prevalence” will be used in the sense of dependence prevalence; if the term is used in a different sense, it will be specified.

Surveys about prevalence tend to underestimate the prevalence of the more damaging and stigmatized forms of drug use. Basically, two methodologies are used to estimate prevalence: direct methods, in which the interviewees are asked if they use or are dependent on cocaine, and the indirect method, when different sources of data are used to estimate prevalence, or interviews are performed, asking the individuals about use or dependence on drugs from their network of contacts. The indirect methods are considered the “gold standard”; however, due to lack of data estimated by indirect methods, only estimates using direct survey methods were included in the modeling^15^.

The GBD 2019 also uses the sociodemographic index (SDI), a measurement resulting from three indicators: total fertility rate, *per capita* income, and average education of the population older than 15 years. The SDI allows comparing data among locations with similar socioeconomic levels^21^. Estimates for 1990 and 2019 will be presented, in addition to percentage variations by sex and age group. For all estimates, 95% uncertainty intervals (95% UI) were presented. The time change was evaluated using the difference between the rates of 1990 and 2019. The differences were considered statistically significant when the 95% UI did not include zero. In this study, all the rates of prevalence, mortality, DALY, YLL, and YLD refer to age-standardized rates per 100,000 inhabitants. The prevalence will be described in absolute numbers.

This study was approved by the Research Ethics Committee of *Universidade Federal de Minas Gerais* (CAAE Project - 62803316.7.0000.5149).

## RESULTS

In Brazil, the age-standardized rate prevalence (ASRP) of MD attributable by cocaine use, in 2019, was 190.28/100.000 (95% UI: 142.29 - 255.93), for both sexes, and it was 2.7-fold higher for men (278.60/100.00; 95% UI: 208.20 - 374.39) when compared to women (104.01/100.000; 95% UI: 76.70 - 143.02). Between 1990 and 2019, there was a positive, statistically significant variation of prevalence of dependence, in absolute numbers (from 204.165 to 436.430) and in the ASRP (from 134.27/100.000 to 190.28/100.000) for both sexes, respectively (Table 1).

**TABLE 1:** Number of cases of dependents and Age-Standardized Prevalence Rate (ASRP) of dependence of mental and behavioral disorders caused by the use of cocaine in Brazil, in 1990 and 2019, and percentage of change from 1990 to 2019, by sex.

	1990		2019		Percentage of chance in the number of cases 1990 to 2019	Percentage of chance in the ASRP 1990 to 2019
	Number of cases	Age-standardized prevalence rate	Number of cases	Age-standardized prevalence rate		
**Male**	135 795	181.34	314 385	278.60	132*	54*
	(97 403 to 188 685)	(132.76 to 246.67)	(234 880 to 422 536)	(208.20 to 374.39)	(105 to 164)	(37 to 73)
**Female**	68 371	89.00	122 045	104.01	79*	17*
	(49 999 to 95 910)	(66.42 to 122.02)	(90 625 to 167 170)	(76.70 to 143.02)	(68 to 93)	(11 to 23)
**Both sexes**	204 165	134.27	436 430	190.28	114*	42*
	(147 448 to 282 872)	(98.91 to 182.28)	(329 015 to 580 991)	(142.29 to 255.93)	(94 to 137)	(30 to 57)

* Differences were considered statistically significant. The values in parentheses are 95% uncertainty intervals (95% UI).

The state of Amazonas showed the highest age-standardized mortality rate (ASMR) in 2019 (1.52/100.000; 95% UI: 1.27 - 1.84), followed by the Federal District (0.60/100.000; 95% UI: 0.50 - 0.73) and Roraima (0.58/100.000; 95% UI: 0.49 - 0.68). The ASMR of MD attributable by cocaine use in Brazil had a positive and statistically significant variation of 278% (95% UI: 245 -321%), between 1990 and 2019. The positive and statistically significant variations in all 27 states is something particularly relevant. The states of Ceará (584%; 95% UI: 394 - 852%), Piauí (554%; 95% UI: 409 - 744%), Tocantins (516%; 95% UI: 374 - 696%), and Paraíba (511%; 95% UI: 385 - 685%) had the greatest variations between 1990 and 2019 (Table 2).

The DALY rates in 2019 were relatively homogeneous among the Brazilian states, except for Amazonas, which had a DALY rate of 113.27/100.000 (95% UI: 92.29 -139.05). The DALY for Brazil was 42.83/100.00 (95% UI: 35.28 - 61.43) - in that, 17.01/100.000 (95% UI: 15.63 - 18.71) for YLL and 25.82/100.000 (95% UI: 15.10 - 39.36) for YLD. Although the YLD was predominant in the composition of the DALY, the increases in YLL during the period were higher in all states when compared to increments in YLD (Table 2).

**TABLE 2:** DALY^a^, YLD^b^, YLL^c^, and ASMR^d^ of mental and behavioral disorders caused by the use of cocaine in Brazil and its states in 2019, and percentage of change from 1990 to 2019.

STATE	ASMR 2019	Percentage of change in the ASMR 1990 to 2019*	DALY 2019	Percentage of change in the DALY 1990 to 2019*	YLD 2019	Percentage of change in the YLD 1990 to 2019*	YLL 2019	Percentage of change in the YLL 1990 to 2019*
Acre	0.48	333	57.20	134	31.83	67	25.37	378
	(0.41 to 0.57)	(245 to 451)	(30.98 to 57.97)	(90 to 200)	(18.58 to 48.55)	(33 to 111)	(20.88 to 30.58)	(271 to 528)
Alagoas	0.20	350	34.47	73	24.18	35	10.29	415
	(0.16 to 0.24)	(250 to 499)	(32.82 to 56.81)	(44 to 116)	(13.67 to 37.46)	(10 to 67)	(8.39 to 12.68)	(295 to 600)
Amapá	0.27	335	37.99	82	23.46	31	14.52	391
	(0.23 to 0.32)	(250 to 438)	(23.54 to 47.81)	(48 to 130)	(13.24 to 36.63)	(6 to 59)	(12.01 to 17.37)	(282 to 525)
Amazonas	1.52	315	113.27	220	30.88	66	82.39	388
	(1.27 to 1.84)	(225 to 424)	(92.29 to 139.05)	(158 to 306)	(17.69 to 48.91)	(33 to 112)	(67.53 to 102.19)	(274 to 538)
Bahia	0.23	131	34.89	55	24.07	35	10.82	134
	(0.19 to 0.28)	(77 to 199)	(34.00 to 63.54)	(31 to 86)	(13.57 to 37.75)	(10 to 67)	(8.58 to 13.18)	(78 to 212)
Ceará	0.19	584	33.82	78	24.02	35	9.80	677
	(0.15 to 0.24)	(394 to 852)	(25.02 to 49.20)	(46 to 126)	(13.67 to 38.12)	(10 to 67)	(7.69 to 12.49)	(451 to 1026)
Distrito Federal	0.60	166	60.18	108	29.59	57	30.60	201
	(0.5 to 0.73)	(109 to 239)	(19.70 to 42.03)	(74 to 152)	(16.99 to 46.06)	(28 to 97)	(24.80 to 37.58)	(133 to 288)
Espírito Santo	0.44	276	52.02	110	29.11	50	22.91	321
	(0.37 to 0.53)	(203 to 361)	(32.37 to 56.00)	(72 to 163)	(16.71 to 44.32)	(22 to 86)	(18.56 to 27.93)	(232 to 427)
Goiás	0.33	232	41.36	85	24.11	35	17.25	284
	(0.26 to 0.40)	(154 to 328)	(29.71 to 56.25)	(53 to 130)	(14.04 to 37.56)	(12 to 67)	(13.53 to 21.57)	(190 to 403)
Maranhão	0.33	232	39.51	75	23.66	32	15.84	232
	(0.26 to 0.40)	(131 to 382)	(27.66 to 51.11)	(44 to 118)	(13.91 to 36.99)	(10 to 62)	(12.43 to 19.76)	(129 to 3.84)
Mato Grosso	0.41	247	47.05	96	26.04	41	21.01	287
	(0.34 to 0.48)	(173 to 348)	(28.90 to 53.79)	(64 to 143)	(14.99 to 40.09)	(13 to 74)	(17.41 to 25.5)	(196 to 434)
Mato Grosso do Sul	0.39	200	43.16	81	23.25	30	19.90	232
	(0.33 to 0.47)	(141 to 266)	(42.93 to 74.84)	(52 to 122)	(13.4 to 36.13)	(5 to 63)	(16.39 to 24.42)	(160 to 311)
Minas Gerais	0.42	348	59.5	156	36.83	92	22.68	469
	(0.35 to 0.51)	(258 to 461)	(28.45 to 54.26)	(107 to 227)	(21.55 to 57.37)	(52 to 144)	(18.45 to 28.00)	(348 to 630)
Pará	0.14	217	29.45	49	22.54	26	6.91	253
	(0.11 to 0.16)	(152 to 291)	(24.38 to 48.42)	(25 to 84)	(12.79 to 34.66)	(4 to 53)	(5.53 to 8.30)	(174 to 349)
Paraíba	0.26	511	39.81	98	26.03	43	13.78	590
	(0.22 to 0.32)	(385 to 685)	(22.24 to 45.64)	(61 to 152)	(14.93 to 40.17)	(16 to 80)	(11.20 to 16.94)	(427 to 812)
Paraná	0.31	285	41.07	91	24.66	37	16.41	372
	(0.26 to 0.37)	(212 to 372)	(43.83 to 80.52)	(58 to 140)	(14.33 to 37.91)	(13 to 68)	(13.27 to 20.02)	(270 to 493)
Pernambuco	0.17	230	32.5	62	23.61	34	8.89	265
	(0.15 to 0.21)	(167 to 311)	(29.69 to 54.56)	(36 to 100)	(13.5 to 37.35)	(8 to 68)	(7.34 to 10.80)	(189 to 369)
Piauí	0.42	554	46.91	127	24.95	40	21.96	675
	(0.35 to 0.50)	(409 to 744)	(25.11 to 48.79)	(82 to 197)	(14.39 to 38.68)	(16 to 73)	(17.76 to 26.70)	(475 to 933)
Rio de Janeiro	0.43	317	48.57	115	25.2	40	23.36	405
	(0.36 to 0.51)	(240 to 407)	(31.13 to 57.59)	(77 to 175)	(14.4 to 38.95)	(14 to 72)	(19.22 to 28.34)	(305 to 526)
Rio Grande do Norte	0.15	430	32.38	71	24.14	37	8.24	541
	(0.12 to 0.19)	(307 to 587)	(44.48 to 73.63)	(41 to 117)	(14.06 to 37.34)	(12 to 70)	(6.45 to 10.47)	(380 to 755)
Rio Grande do Sul	0.32	218	43.01	88	26.83	46	16.19	261
	(0.27 to 0.39)	(163 to 295)	(31.34 to 57.02)	(58 to 129)	(15.24 to 41.47)	(21 to 81)	(13.43 to 19.45)	(192 to 355)
Rondônia	0.32	204	42.77	83	26.3	40	16.47	258
	(0.26 to 0.38)	(134 to 311)	(22.15 to 46.21)	(54 to 126)	(15.24 to 40.53)	(16 to 71)	(13.38 to 20.10)	(168 to 411)
Roraima	0.58	220	57.41	109	27.2	41	30.21	272
	(0.49 to 0.68)	(157 to 302)	(35.25 to 61.92)	(74 to 162)	(15.51 to 43.21)	(12 to 76)	(24.78 to 35.95)	(188 to 390)
Santa Catarina	0.24	210	35.46	94	22.59	52	12.87	262
	(0.20 to 0.29)	(151 to 283)	(36.87 to 62.77)	(63 to 145)	(12.77 to 35.88)	(23 to 93)	(10.43 to 15.98)	(188 to 362)
São Paulo	0.35	244	47.00	73	29.16	29	17.84	326
	(0.29 to 0.42)	(174 to 330)	(23.47 to 47.60)	(45 to 114)	(16.84 to 45.73)	(5 to 56)	(14.67 to 21.77)	(233 to 447)
Sergipe	0.22	337	35.57	78	23.7	34	11.87	419
	(0.18 to 0.27)	(237 to 464)	(45.33 to 77.69)	(47 to 125)	(13.62 to 36.95)	(9 to 63)	(9.50 to 14.56)	(288 to 583)
Tocantins	0.36	516	42.52	105	24.4	34	18.12	600
	(0.29 to 0.43)	(374 to 696)	(38.86 to 68.16)	(67 to 161)	(13.86 to 38.38)	(11 to 64)	(14.66 to 22.25)	(428 to 831)
Brazil	0.32	278	42.83	95	25.82	43	17.01	343
	(0.30 to 0.35)	(245 to 321)	(35.28 to 61.43)	(70 to 128)	(15.1 to 39.36)	(29 to 59)	(15.63 to 18.71)	(299 to 401)

a) DALY: Disability adjusted life years; b) YLD: years lived with disabilities; c) YLL: years of life lost to premature death; d) ASMR: age-standardized mortality rate*.* *Differences were considered statistically significant. The values in parentheses are 95% uncertainty intervals (95% UI).

**FIGURE 1:**
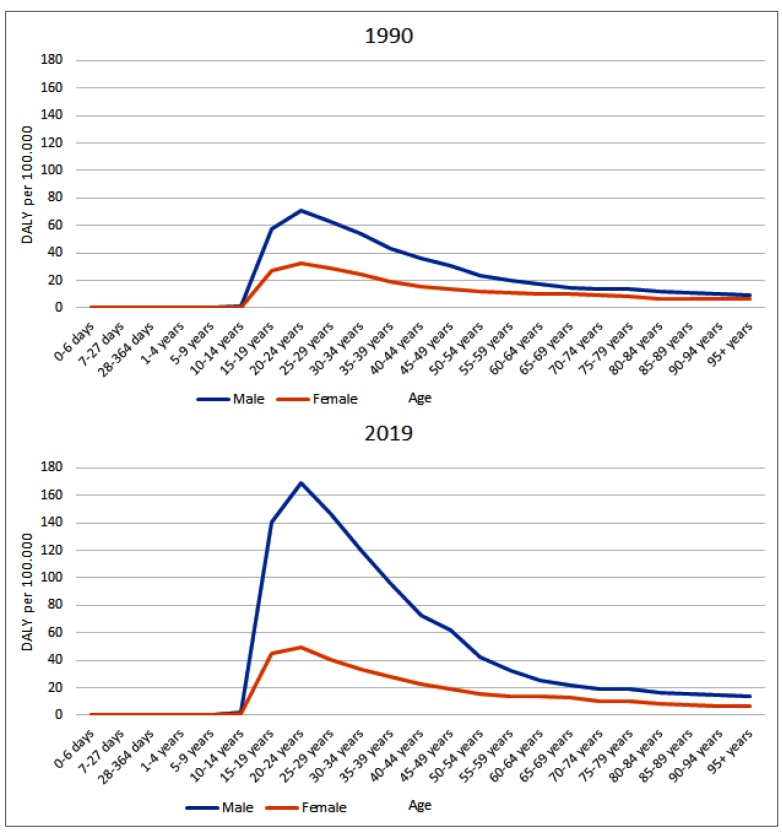
Age-standardized DALY of the mental disorders caused by cocaine use, by sex, in Brazil, 1990 and 2019.

Figure 1 shows the DALY of MD attributable by cocaine use in Brazil between 1990 and 2019, by sex and age group. One can notice that the DALYs were higher in 2019 as compared to 1990, and higher for men than for women, in all age groups. For both sexes, the 20-24-year age group was the most affected by the increase in DALYs. Above that age, the rates decreased progressively.

**FIGURE 2:**
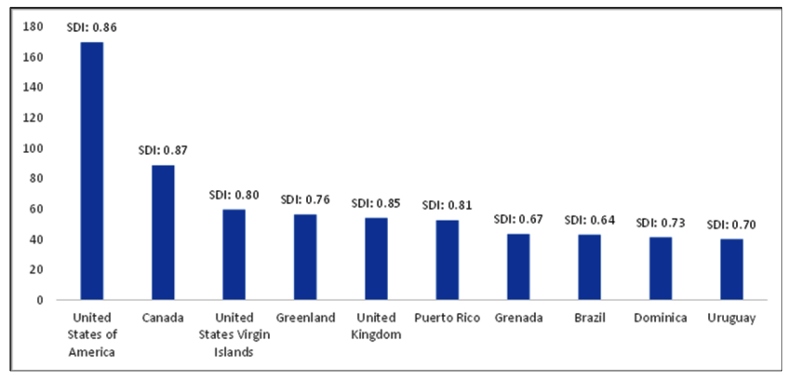
Global ranking of age-standardized DALY of disorders caused by cocaine use and respective sociodemographic Index (SDI), 2019.

In the GBD 2019, among the 204 countries and territories studied, Brazil ranked 8^th^ in DALYs of MD attributable by use of cocaine (42.83 95% UI: 32.37 -56.00), after the USA, Canada, Virgin Islands, Greenland, United Kingdom, Puerto Rico, and Granada (Figure 2).

## DISCUSSION

Brazil has the 8^th^ highest burden of disease attributable to MD attributable by cocaine use in the world, affecting mainly men and young adults. Between 1990 and 2019, the country had a positive, statistically significant variation in the ASRP as well as in the prevalence in absolute numbers. Moreover, the ASMR of MD attributable by use of cocaine had a statistically significant increase in Brazil and in all of its states.

Globally, between 1990 and 2016, the ASRP of MD attributable by use of cocaine showed a decrease, which was higher in women (-7.2%) than in men (-5.8%)^12^. Brazil, on the other hand, had an increase in the ASRP for men and women between 1990 and 2019, by 54% and 17%, respectively.

Moreover, the ASRP verified for Brazil in 2019 (190.28, 95% UI: 142.29 -255.93) was 2.5-fold higher than the global prevalence (77.6, 95% UI: 70.7 - 85.9) in 2016, highlighting the magnitude of the problem of cocaine use in the country. The UNODC^22^ report had already indicated a significant increase in cocaine consumption in Brazil.

In Brazil, the ASRP of MD attributable by cocaine use is 2.68-fold higher among men than among women, confirming historical data indicating the consumption of illicit drugs affects males more often than females^2^ ^,^ ^4^.

In a study by Abdalla *et al*. (2014)^2^, men were 4.4-fold more likely to try cocaine and 4.8-fold more likely to use cocaine than women. However, among female cocaine users, there was a higher proportion of dependents (55%) than among male users (41,4%), which indicates women are more vulnerable and likely to become dependent^3^. In the study conducted by Mesquita et al (2001)^23^, female cocaine users were more likely to have one or more overdoses in comparison to male users.

In Brazil, in 2019, the ASMR (0.3, 95% UI: 0.30 - 0.35) was three times higher than the global estimate from 2017^24^ (0.1, 95% UI: 0.1 - 0.1). Furthermore, a statistically significant increase was found in the ASMR, by 2.78 (95% UI: 2.45 -3.21) in the country, between 1990 and 2019, for both sexes. A Roth *et al* (2018)^24^ argued the increase in ASMR could be partly explained by the poor implementation of harm reduction strategies, and of treatment for drug dependence and abuse on a global scale.

In the study by Bastos and Bertoni (2014)^5^, only a small proportion (1.83%) of the users of crack and similar drugs declared having attended the harm reduction services in the month prior to the survey. However, the increase in ASMR cannot be attributed merely to the lack of health services for drug users; the problems attributable by drugs have, rather, a multifactorial origin, involving biological, psychological, social, economic, and cultural factors^25^.

The rates of YLD (25.82) and DALY (42.83) of MD attributable by cocaine use in Brazil, in 2019, were higher than the global rates in 2016: 10.6 and 15.3, respectively. This confirms the higher magnitude of MD attributable by cocaine use in Brazil, when compared to global data^12^.

There was a major variation in the YLL 343% and it is relevant to note that the most affected are young adults, especially between 20 and 24 years of age, greatly impacting the YLL and YLD. The composition of the 2019 DALY for MD attributable by cocaine use in Brazil was predominantly related to disability (YLD) and less to premature death (YLL). However, the YLL between 1990 and 2019 (343%) was eight-fold higher than the YLD (43%), indicating an increase in early mortality attributable by MD attributed to use of cocaine use. 

One must also consider the estimates of the burden of illicit drugs in the GBD 2019 might be considered too conservative, since several other possible health problems related to drug use^6^ ^,^ ^12^ were not included in the GBD 2019 estimates and the study data are underestimated. In Brazil, a large proportion of violence corresponds to murders by firearms related to drug trafficking, availability and presence of illicit weapons, and consumption of alcohol and drugs^25^ ^-^ ^27^. The use of crack cocaine is significantly associated with an increased risk of violent deaths^6^. In a cohort study, which followed up 131 crack users for five years, in the state of São Paulo, the top causes of death were homicide and AIDS^6^. In addition, people who reported lifetime use of cocaine had an elevated mortality risk due to external causes (poisoning, suicide, homicide, and unintentional injury) and to infectious diseases^28^. Moreover, a study indicated alcohol and cocaine consumption were significantly associated with victims of violence^26^.

When compared to 1990, DALYs in 2019 were higher for all age groups, for men and women. Bonadiman *et al*. (2017)^29^ indicated MD attributable by use of illicit drugs in Brazil had a higher increase in DALY rates than all other MDs studied in the period of 1990 to 2015 (37.1%). The age and sexual pattern in the DALYs of MD attributable by cocaine use corroborate data from the profile of cocaine and crack users in the country, affecting mostly the young (including children and adolescents) and males^2^ ^,^ ^5^.

Worldwide, in 2016, the burden of MD attributable by illicit drug use was higher in the countries with a higher SDI^12^. However, against that trend, Brazil (SDI: 0.64) has one of the 10 highest burdens of disease associated with use of cocaine in the world. In a systematic revision, it was noticed the low cost of crack cocaine promoted its spreading and consumption among poor and destitute segments of the population^30^.

There was little variability in the estimates of DALY and YLD among the states of Brazil, regardless of the country extension, and cultural and socioeconomic differences. However, the state of Amazonas stands out for its high rates of ASMR, DALY, and YLL in comparison to other states. One hypothesis is that the state of Amazonas has a higher proportion of “garbage codes”^31^, and the redistribution provided by the GBD may have increased those estimates.

In Abdalla *et al*. (2014)^2^, the rates of prevalence of cocaine use in the last year varied among the Brazilian regions, ranging from 1.8% to 2.6%, with the lowest rates in the South region. The Southeast and Midwest had the highest rates of experimentation and of use when compared to other regions.

In Brazil, epidemiological data on the prevalence of drug use provided by studies are still scarce^2^ ^,^ ^4^, and are employed as the source for the estimated YLD. The YLL, however, is based on mortality databases, which are annually updated and showed significant differences in growth and among the states.

One limitation in all estimates of causes of death by drug use was the variation, among countries, of the ICD-10 codes used to classify overdose - some counties have additional codes, which allows for a more precise definition of the cause of death associated with specific substances, while others do not^12^. In addition, a brazilian study discusses the difficulty of working with deaths from intoxication using the ICD-10, since the classification of these deaths by toxic agent is poorly qualified, which leads to underestimation of cases and reduces their reliability^32^. Another limitation that also leads to mortality underestimation is the use of SIM database, despite being a qualified system, there are differences between brazilian states, both in coverage and in data quality^33^.

In 2019, there was an important methodological improvement in the GBD concerning the redistribution by algorithms of different garbage codes related to drug use, by applying specific methods for Brazil, and considering the evidence of high cocaine consumption in the country (one of the highest in the world), which provided better adjustment and improvement in the estimates^17^. The GBD results are an important evaluation tool of the MD attributable by cocaine use in the country, providing data that may contribute to definition of public policies. Other adverse effects of the use of illicit drugs on society (in terms of social, family, and economic problems) should be studied in greater depth, using other methods.

Brazil stands out in the world for the highest DALYs of MD attributable by cocaine use and shows high and statistically significant increases in the ASMR at national level and in its states. It is important to emphasize the mortality and burden attributed to use of drugs is considered avoidable and modifiable. These results demonstrate the need for new public policy strategies to fight against drug consumption in Brazil.

Understanding the patterns of ASMR, prevalence, and DALY help to qualify the prevention and treatment strategies, which must take into consideration the characteristics of those indicators, to implement early intervention strategies for populations and regions that need them the most.
